# Role of dendritic cells in MYD88-mediated immune recognition and osteoinduction initiated by the implantation of biomaterials

**DOI:** 10.1038/s41368-023-00234-3

**Published:** 2023-08-02

**Authors:** Zifan Zhao, Qin Zhao, Hu Chen, Fanfan Chen, Feifei Wang, Hua Tang, Haibin Xia, Yongsheng Zhou, Yuchun Sun

**Affiliations:** 1 Center of Digital Dentistry, Faculty of Prosthodontics, Peking University School and Hospital of Stomatology & National Center for Stomatology & National Clinical Research Center for Oral Diseases & National Engineering Research Center of Oral Biomaterials and Digital Medical Devices & Beijing Key Laboratory of Digital Stomatology & Research Center of Engineering and Technology for Computerized Dentistry Ministry of Health & NMPA Key Laboratory for Dental Materials, Beijing, China; 2 The State Key Laboratory Breeding Base of Basic Science of Stomatology (Hubei- MOST) & Key Laboratory of Oral Biomedicine, Ministry of Education, School & Hospital of Stomatology, Wuhan University, Wuhan, China; 3 Institute of Infection and Immunity, Medical Science and Technology Innovation Center, Shandong First Medical University & Shandong Academy of Medical Sciences, Jinan, China

**Keywords:** Stem cells, Nuclear receptors

## Abstract

Bone substitute material implantation has become an important treatment strategy for the repair of oral and maxillofacial bone defects. Recent studies have shown that appropriate inflammatory and immune cells are essential factors in the process of osteoinduction of bone substitute materials. Previous studies have mainly focused on innate immune cells such as macrophages. In our previous work, we found that T lymphocytes, as adaptive immune cells, are also essential in the osteoinduction procedure. As the most important antigen-presenting cell, whether dendritic cells (DCs) can recognize non-antigen biomaterials and participate in osteoinduction was still unclear. In this study, we found that surgical trauma associated with materials implantation induces necrocytosis, and this causes the release of high mobility group protein-1 (HMGB1), which is adsorbed on the surface of bone substitute materials. Subsequently, HMGB1-adsorbed materials were recognized by the TLR4-MYD88-NFκB signal axis of dendritic cells, and the inflammatory response was activated. Finally, activated DCs release regeneration-related chemokines, recruit mesenchymal stem cells, and initiate the osteoinduction process. This study sheds light on the immune-regeneration process after bone substitute materials implantation, points out a potential direction for the development of bone substitute materials, and provides guidance for the development of clinical surgical methods.

## Introduction

Bone substitute material implantation has become an important treatment strategy for the repair of oral and maxillofacial bone defects. The success of this technique is dependent on the induction of osteogenesis and optimal regulation of the local immune microenvironment.^1–4^ Traditionally, inflammatory reactions and immune cells have been considered as adverse factors affecting the biological effects of implanted materials. Therefore, one of the most popular research topics in this field is the development and application of biomaterials that minimize the immune response and the resulting inflammatory effect in the local microenvironment.^5–9^ However, according to a recently emerged view in the field of osteoimmunology, the crosstalk between implantable bone substitute materials and the immune system plays an important positive role in osteogenesis.^10,11^ That is, the immune balance maintained by immune cells determines the final fate of biomaterials in the complex in vivo environment. Excessive immune response and insufficient immune response both may lead to failure of osteoinduction.^12–16^ As a widely used implantable bone substitute material, Biphasic calcium phosphate (BCP) has been proven to have osteoinductive effect by many studies.^17,18^ Our previous studies showed that in insufficient inflammatory environment, such as minimally invasive surgery implantation or T cell defects, will lead to failure of osteoinduction.^12^ This result shows that appropriate inflammatory response, including adaptive immune response, is essential to the osteoinduction of BCP. However, as a non-antigenic material, how BCP activates adaptive immunity is still unclear.

The adaptive immune response is considered to be activated by the antigen recognition function of DCs.^19,20^ The function of antigen recognition by DCs in the case of tumors or infection is obvious, but it is unclear in the case of BCP materials that do not have antigens.^21,22^ As a result, the mechanism through which implantable biomaterials are recognized by the immune system and lead to activation of the adaptive immune response remains unclear. In particular, the mechanism by which DCs recognize biomaterials and initiate osteogenesis is still in the preliminary research stage.^20,23,24^

DCs are capable of recognizing intracellular proteins released by necrotic cells through damage-associated molecular patterns (DAMPs).^25,26^ One of these proteins is HMGB1, which is widely distributed in the liver, brain, lungs, heart, spleen, kidneys, and lymphoid tissue.^27^ HMGB1 is released in response to necrosis caused by tissue injury that can be recognized by TLR4 on DCs and activate the MYD88 signaling pathway.^28–31^ However, in trauma tissue, HMGB1 is cleared in a short time, and as a result, it is unable to activate the immune response for a long time.^28^ Based on these findings, in the current study, we hypothesize that HMGB1 is adsorbed onto biomaterials, where it is retained and amplifies the danger signal enough to activate recognition by DCs. This may represent an essential link in the process of osteoinduction. Thus, we focus on the positive regulation of the immune response in the process of osteoinduction and explore the complete immune-regeneration process that occurs after the implantation of biomaterials. In addition, this study reveals the key roles of DCs in the process of osteoinduction mediated by bone substitute materials, and points out a potential new direction for the development of biomaterials.

## Results

### Role of DCs in the osteoinduction effect of BCP

**Fig. 1:**
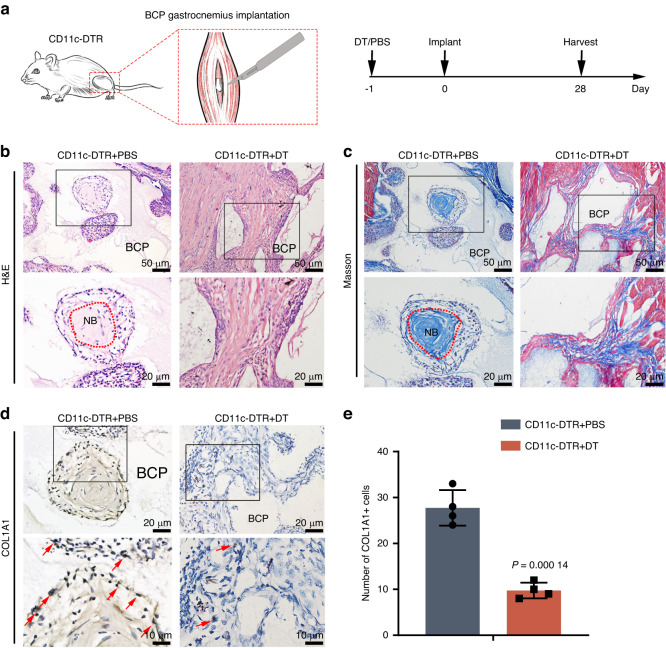
Effect of DC depletion on the osteoinduction ability of BCP. **a** Schematic diagram of a transgenic mouse model. **b**, **c** H&E and Masson’s staining showed the formation of new bone (NB), indicated by the red dotted line. **d** IHC of COL1A1 showed expression of the osteogenesis indicator. Some representative positive cells are indicated with red arrows. **e** Comparison of quantitative COL1A1 expression (*n* = 4, *P* = 0.000 14)

To confirm the role of DC in the osteoinduction of BCP, we constructed BCP muscle implantation model in DC-deficient mice. On the day before implantation, CD11c-DTR mice in the experimental group were administered DT, and the control group of CD11c-DTR mice were administered PBS. Gastrocnemius tissue samples were harvested for histological staining at 28 days after implantation (Fig. 1a). H&E and Masson’s staining showed that there was significant new bone formation (yellow area) in the CD11c-DTR + PBS group 4 weeks after BCP implantation, while no new bone formation was detected in the CD11c-DTR + DT group (Fig. 1b, c). IHC staining revealed significantly lower expression of the osteogenesis marker COL1A1 in the CD11c-DTR + DT group than in the CD11c-DTR + PBS group (*P* = 0.000 14) (Fig. 1d, e). These results indicate that the deletion of DCs directly hinders the osteoinduction effect of BCP.

### Peak in the number of DCs at 4 days after BCP implantation

We used IF staining and flow cytometry to explore the interactions between DCs and BCP over time, that is, within 1–14 days after the implantation of BCP in WT mice. Immunofluorescence staining showed that DCs were present in the tissues around BCP in the BCP-treated mice (Fig. S2a), but very few DCs were observed in the sham-operated tissues without BCP implantation (Fig. S2b). Further, flow cytometry was performed for quantitative evaluation of DCs, and the results showed that the number of DCs reached a peak at 4 days (Fig. S2c, d). Therefore, further evaluations of the mechanism via which BCP activates DCs were performed at the 4-day time point after implantation.

### Trauma-related BCP surface adsorbates are the key to activate DC

**Fig. 2:**
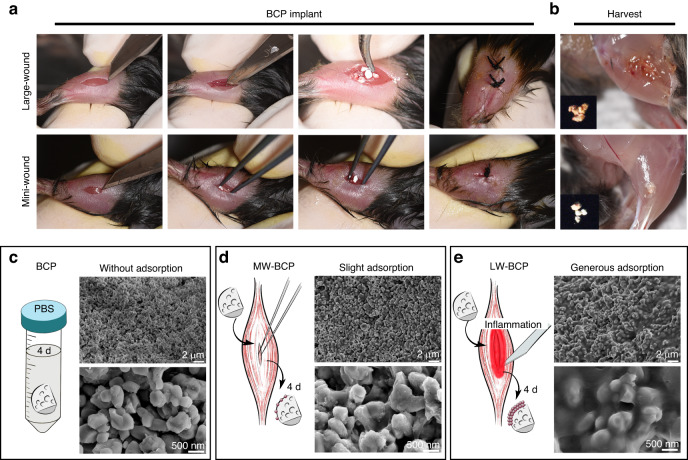
Association of the adsorption substance on the surface of implanted BCP with the severity of implantation trauma. **a**, **b** Procedure for BCP implantation and harvest. **c**–**e** Diagram and SEM image of **c** PBS-treated BCP, **d** MW-BCP, and **e** LW-BCP

To confirm whether BCP-activated DC is related to implantation surgery trauma, we subjected the BCP granules to various treatments as follows. First, BCP granules soaked in PBS for 4 days were used to simulate the changes caused by degradation. In the next two treatments, trauma-related factors were evaluated by mini-wound implantation (MW-BCP) or large-wound implantation (LW-BCP) of BCP granules for 4 days (Fig. 2a, b). First, SEM was used to detect the micromorphology of the above groups of BCP granules. The observations indicated that the micro-porosity of the BCP surface in the implantation groups (Fig. 2c) was not significantly different from that of the PBS-treated BCP (Fig. S1b). Surprisingly, the findings indicated that the granules in the LW-BCP and MW-BCP groups had an “adsorption substance” on their surface, and the amount of the adsorption substance was significantly associated with the severity of implantation trauma (Fig. 2d, e).

**Fig. 3:**
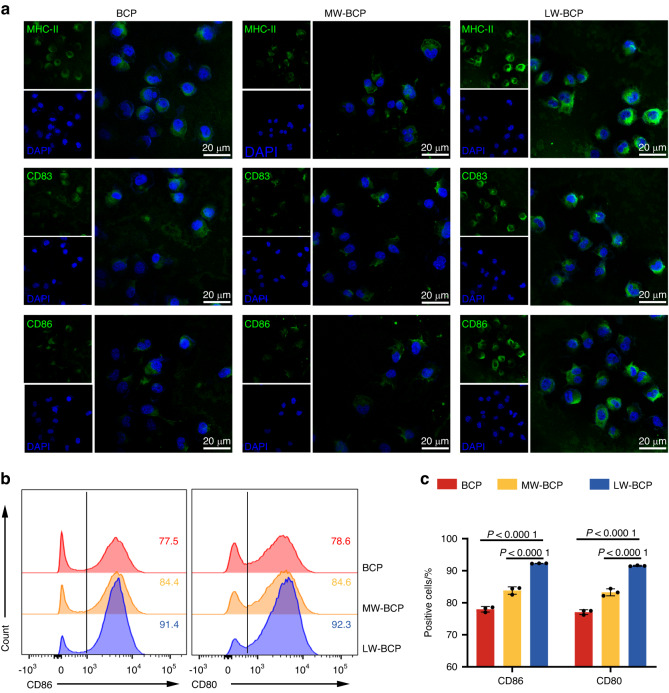
Association of the DC activation ability of BCP with the severity of implantation trauma. **a** Immunofluorescence staining of MHC-II, CD83, CD86 (green), and nuclei (blue). **b**, **c** Flow cytometry and semiquantification of DC activation markers CD80 and CD86 (*n* = 3). DCs were cultured with PBS-treated BCP, MW-BCP, or LW-BCP for 24 h

To further explore the effect of the adsorption substance, we stimulated DC2.4 cells for 24 h with an eluent of PBS-treated BCP, MW-BCP, and LW-BCP granules to detect DC activation. The immunofluorescence (Fig. 3a) and flow cytometry (Fig. 3b, c) results indicated that, compared with the PBS-treated BCP group, expression of the DC activation markers CD80, CD83, and CD86 was significantly upregulated under LW-BCP stimulation, but not under MW-BCP stimulation. This result confirmed that the ability of BCP granules to activate DCs is clearly related to the severity of the implantation trauma, which also seems to be associated with the amount of adsorption substance present on the surface of the material.

### Role of the danger signal protein HMGB1 produced by implantation trauma in triggering immune recognition

**Fig. 4:**
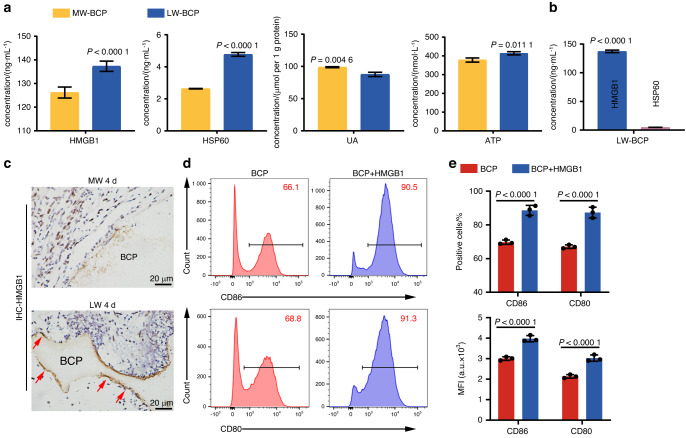
Adsorption of HMGB1 onto the surface of BCP and activation of DCs. **a** ELISA of danger signal proteins in the PBS-treated BCP, LW-BCP, and MW-BCP eluents (*n* = 3). **b** ELISA of HMGB1 and HSP60 in muscles (*n* = 3). **c** IHC staining showed that HMGB1 had adsorbed onto the surface of BCP (marked by red arrows). **d**, **e** Flow cytometry and semiquantification of DC activation markers CD80 and CD86 (*n* = 3)

In order to identify which danger signal proteins, play a role in DC activation in BCP-implanted muscle, we prepared eluates of LW-BCP and MW-BCP and detected the concentrations of various danger signal proteins by ELISA. Significant differences were detected in the concentrations of HMGB1 and HSP60 between the LW-BCP and MW-BCP groups, but not there were no significant differences in ATP or UA (Fig. 4a). Further, the HSP60 concentration was significantly lower than the HMGB1 concentration (Fig. 4b). These results suggest that HMGB1 is probably the most critical molecule for immune activation by implantation biomaterials. We confirmed this by immunohistochemical staining for HMGB1 and found that HMGB1 was indeed present in the adsorption substance on the surface of BCP, but it was not detected in the MW-BCP group (Fig. 4c). Furthermore, DCs were stimulated with BCP or BCP + HMGB1 (5 µg·mL^−1^) for 24 h. The flow cytometry results confirmed that BCP soaked in HMGB1 resulted in significantly greater activation of DCs (*P* < 0.000 1) (Fig. 4d, e).

In the sham-operated model, HMGB1 could not be detected by immunohistochemistry; this is probably because it was eliminated rapidly in the extracellular matrix (Fig. S3). The sham operation involved incision without implantation of BCP (Fig. S2b), and the lack of recognition of DCs can be regarded as a self-protection mechanism.

### Role of the TLR4/MYD88/NF-κB axis in the recognition of biomaterials by DCs

**Fig. 5:**
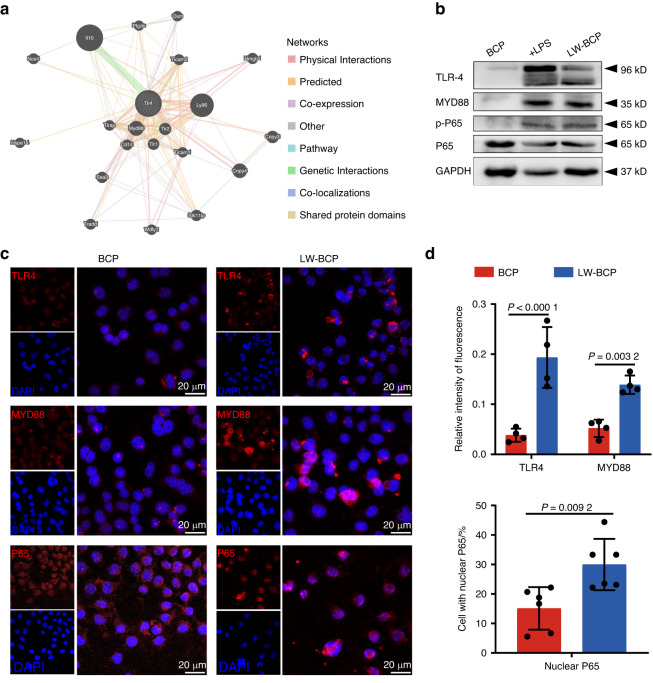
Recognition of biomaterials by DCs via the TLR4/MYD88/NF-κB signaling axis. **a** Co-expression of the HMGB1-associated TLR4-Myd88 pathway with the GENEMANIA database. **b** Western blots showing expression of proteins in the TLR4/MYD88/NF-κB signaling axis for each group. DC + LPS as a positive control group of signal pathway activation. **c** Immunofluorescence staining of TLR4, MYD88, P65, and nucleus. **d** Quantification of positively stained cells or nuclear p65 (*n* = 3)

Protein-protein interaction analysis in the present study indicated that HMGB1 mainly activates the immune recognition process through the TLR4-MYD88 signaling pathway (Fig. 5a). Therefore, we tried to determine whether the TLR4/MYD88/NF-κB signaling axis plays an equally important role in recognition of biomaterials by DCs. To this end, we used PBS-treated BCP, LW-BCP, and MW-BCP eluents to induce DC2.4 cells and observe the activation of signaling pathways. Lipopolysaccharide was used as a positive control for activation of the TLR4/MYD88/NF-κB signaling pathway. Western blot analysis showed that the TLR4/MYD88/NF-κB signaling axis was significantly upregulated in cells treated with the LW-BCP eluent (Fig. 5b). The results of immunofluorescence analysis were similar, as the expression of TLR4 and transmembrane protein MYD88 and nuclear NF-κB-p65 expression were significantly upregulated (Fig. 5c, d). These results suggest that HMGB1 is adsorbed onto biomaterial surfaces, where it activates DCs through the TLR4/MYD88/NF-κB signaling pathway.

### Activated DCs promote MSCs recruitment rather than osteogenic differentiation

**Fig. 6:**
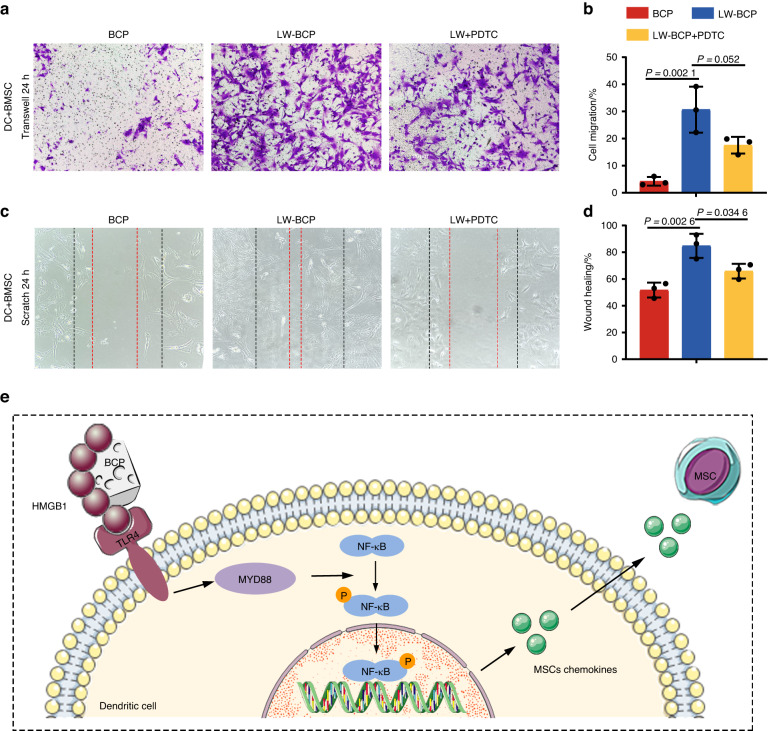
Promotion of MSC recruitment by activation of the TLR4/MYD88/NF-κB signaling axis. **a**, **b** Representative Transwell migration assay images and quantification of each group (*n* = 3). **c**, **d** Representative scratch test images and semiquantification of each group (*n* = 3). **e** Schematic diagram depicting the recognition of biomaterials by DCs through the TLR4/MYD88/NF-κB signaling axis and recruitment of MSCs. PDTC is an inhibitor of the NF-κB signal pathway

We treated DCs with BCP and LW-BCP eluents, then co-cultured treated DCs with primary BMSCs. Transwell migration assays showed that BMSC recruitment was significantly higher in the LW-BCP group than in the BCP group (Fig. 6a, b), and the scratch test revealed similar results (Fig. 6c, d).

Further, we treated DCs with PDTC, an inhibitor of the NF-κB signaling pathway, to block DC activation in the LW-BCP group. The results showed that blocking of the NF-κB signaling pathway led to a significant decrease in the ability of activated DCs to recruit BMSCs (Fig. 6a–d). Under the same conditions, we also induced the mineralization of BMSCs (by adding the culture supernatant of different groups of DCs to a BMSC mineralization culture system). After 14 days of mineralization induction, staining for ALP activity was used to detect the degree of mineralization. The results showed that there was no significant difference in the degree of mineralization of BMSCs between the groups (Fig. S4a). Western blots of the mineralization of related proteins also revealed similar results, that activated DCs did not significantly promote the expression of mineralization-related proteins in BMSCs (Fig. S4b). The above experimental results indicate that activated DCs participate in the process of bone regeneration by promoting the recruitment of MSCs, rather than by directly inducing the osteogenic differentiation of MSCs (Fig. 6e).

### Role of MYD88-mediated immune recognition in the osteoinduction ability of BCP

**Fig. 7:**
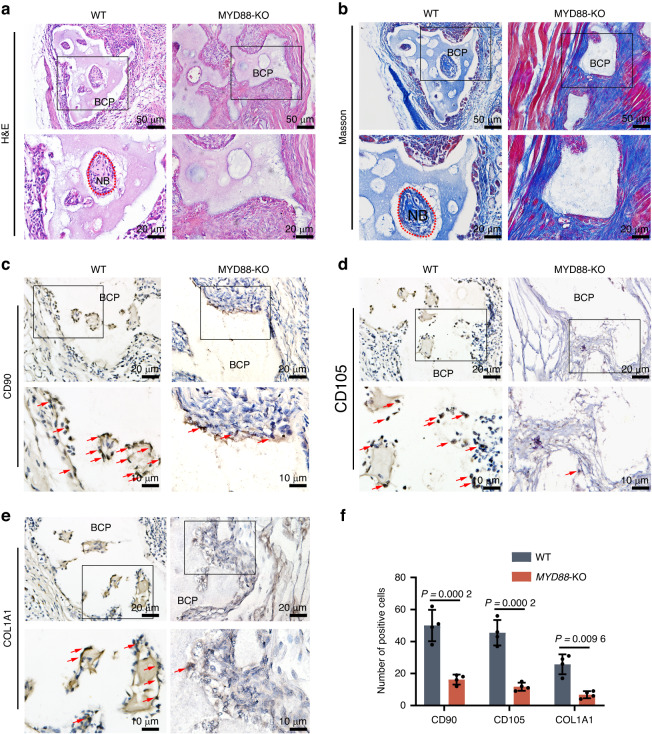
Significant reduction in the osteoinduction and MSC recruitment abilities of BCP in *MYD88*-KO mice. **a**, **b** H&E and Masson staining showed the formation of new bone (NB), indicated with the red dotted line. **c**–**e** IHC of the MSC markers CD90 and CD105 and the osteogenic indicator COL1A1. Some representative positive cells are indicated with red arrows. **f** Comparison of quantitative expression (*n* = 4)

**Fig. 8:**
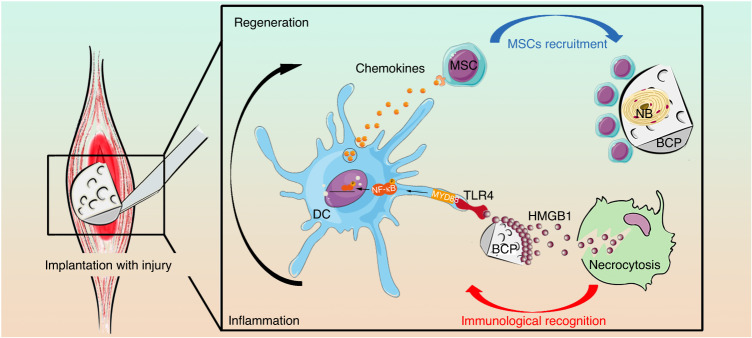
After implantation, HMGB1 adsorbed on the BCP surface was recognized by DCs-TLR4/MYD88/NF-κB signal axis, initiating immune and regeneration processes

Finally, *MYD88*-KO mice were used to verify the importance of the DC-mediated immune recognition signaling pathway in the process of osteogenesis. H&E and Masson staining showed that new bone formation was not observed in *MYD88*-KO mice at 4 weeks after BCP implantation (Figs. 7a, b and 8). We used IHC staining of COL1A1, CD105, and CD90 to observe the recruitment of MSCs in the *MYD88*-KO mice. COL1A1 is a marker of osteogenically differentiated MSCs, and CD105 and CD90 are MSC markers. The results confirmed that the number of overall MSCs or osteogenically differentiated MSCs around BCP in the *MYD88*-KO mice was significantly lesser than that in WT mice (Fig. 7c–f). These results confirm that MYD88-mediated DC immune recognition is necessary for BCP osteoinduction.

## Discussion

The interaction between the immune system and tissue regeneration process has been attracting a lot of attention, and optimal immune regulation has become the main focus in the development of bone substitute materials. BCP has been widely proven to have an osteoinductive ability to form an ossification structure in muscle, but any factor unfavorable to ossification in immune response may lead to osteoinduction failure.^32,33^

In addition to the fact that innate immune cells such as macrophages have been confirmed to participate in the process of osteoinduction.^34^ In recent years, the role of DC in tissue engineering has also received increasing attention.^35,36^ Previous studies have confirmed that dendritic cells, as new participants in bone immunology, interact with biomaterials to promote their biological effects.^35^ More accurate evidence is that studies have found that dendritic cells can promote tissue regeneration by mediating the recruitment of mesenchymal stem cells through extracellular vesicles.^36^ In order to explore the role of DCs in the osteoinduction process of BCP, we chose DC-deficient CD11c-DTR mice, as they are widely used for implantation studies.^37–39^ CD11c is a marker of mature DCs, and it is expressed by pDCs and cDCs, among other DC subsets.^40^ Research has found that administering DT in CD11c-DTR mice can eliminate mature DCs. Compared to the congenital absence of DC, this method has a milder and even negligible impact on the development of other parts of the body.^41,42^ We have successfully observed that BCP in DC-deficient mice has no osteoinductive effect. This confirms that DCs were essential for BCP osteoinduction. As one of the basic coordinators of the immune response, DCs often mature and aggregate in the focus area in the middle and early stages after a stress response such as infection and injury.^43,44^ Similarly, using flow cytometry and immunofluorescence, we observed that the DCs around BCP reached a peak 4 days after implantation, at which time BCP has the ability to activate DCs. Therefore, BCP was taken out on the 4th day after implantation for in vitro experiment.

Current research on materials that activate the immune system mainly focuses on their macroscopic properties (e.g., roughness and hydrophilicity)^45,46^ and surface microstructure.^34^ However, the findings of in vivo and in vitro experiments differ even for the same materials. For example, in our previous study, we found that BCP could not activate DCs under in vitro conditions,^8^ but it could recruit and activate DCs under in vivo conditions (that is, when implanted). This could mean that the recruitment and activation of DCs by BCP after implantation is dependent on the in vivo environment. That is, the in vivo environment may affect the surface morphology of biomaterials, including their surface porosity^47–49^ or cause changes in the physical arrangement of molecular building blocks.^50^ Further, the initial reaction caused by the implantation wound may also endow the material with unique biological properties.^51^ To this end, PBS immersion was used to simulate the degradation process of BCP, and large or mini-wound implantation were used to change the initial reaction of the implantation wound.

The results of this study showed that BCP implanted in large wounds can activate DCs.

Impaired clearance of injury or apoptotic cells leads to the pathological accumulation of necrosis and the release of “danger signals” that are recognized by DC surface receptors and subsequently induce an inflammatory response that initiates tissue repair.^26,52,53^ At present, HMGB1, ATP, UA, and HSP are the main trauma-related danger signal proteins that can be recognized by the DC surface receptors TLR4,^54,55^ DNGR-1,^26^ and TLR3/7/8.^56,57^ These results corroborate previous results which demonstrate that HMGB1 played a role in BCP-induced activation of DCs.^58^ As an endogenous autoantigen released by cells, HMGB1 is a danger signal that can be recognized by DAMPs.^59^ During the inflammatory reaction in the early stages of injury, there are abundant ROS in the extracellular matrix, and they lead to the formation of a disability bond of HMGB1 that activates TLR2 and TLR4 and causes the release of pro-inflammatory chemokines and cytokines that subsequently activate innate and adaptive immunity.^60^ The results of this study showed that HMGB1 adsorbed more on the surface of BCP than other DAMPs and was more related to implantation trauma.

The next question we sought to answer was whether the activation of DCs by danger signal proteins, such as HMGB1, is dependent on the presence of biomaterials. In other words, we wanted to clarify the role of biomaterials in this process. Another important question was whether the activation of DCs by danger signal proteins was sufficient and persistent.^61^ In Supplementary Figs. 3, 2b, no extracellular HMGB1 and activated DC were detected. As a scaffold material, BCP provides HMGB1 with medium and space for adsorption, which is more difficult to be eliminated than in the extracellular matrix. These findings indicate that the presence of BCP is essential for the recognition of HMGB1 by DCs. As BCP has good adsorption ability on account of its porous structure, this characteristic is probably useful for the aggregation of HMGB1 and other danger signal proteins that eventually lead to a series of inflammatory and regeneration reactions.

This study found that TLR4/MYD88/NF-κB signaling axis is the main signal pathway for DCs to recognize HMGB1. Similar to the results of this study, several receptors of DCs related to DAMPs have been identified, such as TLR4, which mainly acts as a receptor of HMGB1, HMGN-1, HSP, and other self-molecules. The TLR4/MYD88/NF-κB signaling axis has been proven to be of great significance in self-tumor cell clearance and autoimmune diseases.^62,63^ PDTC were selected as the NF-κB inhibitor, which can inhibit the phosphorylation of IκB, and prevent the translocation of NF-κB into the nucleus.^64^ MYD88 is the canonical adapter for inflammatory signaling pathways that is present downstream of the TLR and IL-1 receptor families, and it is the central node of the inflammatory pathway.^65^ Therefore, we used *MYD88*-KO mice for BCP implant experiments, as they are widely used for such studies.^66–68^

At last, we explored the mechanism by which DCs participate in the regulation of osteogenic induction after they are activated by biomaterials. Previous studies have reported that DCs activated the adaptive immune response dominated by activated T cells after antigen activation and presentation, and can participate in the recruitment of MSCs.^12,69^ However, we need to determine whether the promotion of osteogenesis by DC activation is dependent on the adaptive immune response. It is known that immune cells play a dual role in osteogenesis by regulating the osteogenic differentiation of MSCs^70^ and the recruitment of MSCs in the bone regeneration area.^71^ The results of this study show that activated DCs contribute to the recruitment of MSCs, but have no obvious effect on the osteogenic differentiation of MSCs. Similar to our experimental results, it has been reported that DCs can release chemokines to recruit MSCs, and that DCs can also mediate the recruitment of MSCs by releasing outer vesicles under in vitro conditions.^36,72^ Up to now, there have been very few studies on the regulation of the osteogenic differentiation of MSCs by DCs, so these findings are valuable. It is worth noting that recent studies have shown that the complexes formed by implanting biomaterials into the body that can activate DC immune recognition are called biomaterial-related molecular patterns (BAMPs), similar to DAMPs. The research results presented in this study provide evidence for BAMPs, but their structure needs further exploration in future research.^73,74^

In conclusion, after BCP implantation, danger signal proteins such as HMGB1 aggregate and are adsorbed onto the surface of BCP, are recognized by the TLR4/MYD88/NF-κB signaling axis of DCs, and recruit MSCs into the microenvironment around materials to initiate and promote the process of osteoinduction (Fig. 8). The findings of this study shed light on the mechanism by which non-antigen biomaterials activate the body’s natural and adaptive immunity, as well as complement and improve the immune and regeneration process. This study emphasizes that DCs were indispensable for the osteoinduction process of BCP. This discovery provides a new idea for the research, development, and application of bone substitution materials.

## Methods and materials

### Preparation and characterization of BCP

BCP granules were prepared by wet chemical precipitation according to a previously described method.^12,75,76^ The BCP granules were prepared at a HA/β-TCP ratio of 60/40, with a diameter of about 0.6 mm. The porosity and specific surface area of all granules are uniform.

X-ray diffraction (XRD) and scanning electron microscopy (SEM) were respectively used to analyze the elemental composition and surface morphology of BCP granules (Supplementary Fig. 1).

### Animals and ethical approval

BCP implantation were performed in 8-week-old female C57BL/6 wild type (WT) or transgenic mice. CD11c-DTR mice and *MYD88*-KO mice were obtained from Jackson Laboratory. Details are as follows. CD11c-DTR: https://www.jax.org/strain/004509, MYD88-KO: https://www.jax.org/strain/009088.

The DC-knockout group and control group mice were injected with diphtheria toxin (DT, 100 ng/mice, Sigma, USA) and isopyknic phosphate-buffered saline (PBS), respectively, 1 day before BCP implantation (Fig. 1a).

All the mice were treated according to the ethical guidelines of the Laboratory Animal Welfare Ethics Branch of the Biomedical Ethics Committee of Peking University (approval number LA2022320).

### Implantation of BCP in the gastrocnemius muscle

Briefly, for large-wound implantation (LW), an 8-mm incision was made through the skin and muscle, 2.5 mg BCP granules were implanted into the muscle incision. For mini-wound implantation (MW), the muscles were directly separated with micro tweezers, and 2.5 mg BCP granules were implanted into a 2-mm gap to model implantation. The implantation and harvest process are shown in Fig. 2a, b.

### Histological staining

After harvest the implant with surrounding gastrocnemius tissue, the samples were immersed for 3 weeks in Ethylene Diamine Tetraacetic Acid (EDTA) decalcifying solution that was replaced every two days for decalcification. The formula of EDTA decalcifying solution is as follows: EDTA-2Na: 200 g, NaH_2_PO_4_-2H_2_O: 25.28 g, Na_2_HPO_4_-12H_2_O: 13.6 g, NaCl: 18 g, NaOH: 25.5 g, ddH_2_O: 2 000 mL. The samples were then dehydrated with gradient ethanol, and paraffin sections were prepared. The sections were subjected to hematoxylin and eosin (H&E), Masson, immunohistochemical (IHC), and immunofluorescence (IF) staining. For IHC and IF staining, sections were treated with primary antibodies against CD11c, COL1A1, HMGB1, CD90, and CD105 diluted to 1:200 (Abclonal, China).

### Cell culture

Mouse primary bone mesenchymal stem cells (BMSCs) and DC2.4 cells were cultured in Dulbecco’s Modified Eagle Medium (DMEM; Hyclone, USA), containing 10% fetal bovine serum (FBS; Hyclone, USA) and 100 U·mL^−1^ Pen&Strep (Gibco, USA) at 37 °C under humid conditions in a 5% CO_2_ atmosphere. Pyrrolidine dithiocarbamate (PDTC,10 μmol·L^−1^, Selleck, USA) was used to block the NF-κB signaling pathway.

### Transwell migration assays

Mouse BMSCs were cultured under starvation for 12 h in 2% FBS DMEM, seeded in 24-transwell chambers with 5 000 cells per chamber, and co-cultured with three groups (BCP, LW-BCP, and LW + PDTC) of DC2.4 cells for 24 h. The cells were then stained with crystal violet (Beyotime, China).

### Scratch test

DC2.4 supernatants from the three groups were added to the starvation culture (described in 2.5.2) of mouse BMSCs for 24 h, and microscopic photographs were obtained.

### Osteogenic induction and alkaline phosphatase staining

BMSCs were cultured with supernatants from the three groups (BCP, LW-BCP, and LW + PDTC) of DCs in an osteogenesis-inducing medium, which contained 10 nmol·L^−1^ dexamethasone, 10 mmol·L^−1^ β-glycerophosphate, and 50 μg·mL^−1^ L-ascorbic acid, and was renewed every two days. Alkaline phosphatase (ALP) staining was performed according to the protocol described by the manufacturer of the ALP staining kit (Beyotime, China).

### Preparation of BCP eluent

BCP granules (0.5 g) from each group (BCP, MW-BCP, and LW-BCP) were immersed in PBS and shaken at 37 °C for 24 h. Suction filtration of the solution through a 70-μm filter was performed, and the eluent was used for molecular detection and cell experiments.

### Flow cytometry

Cells were incubated with CD80-PE (1:400), CD86-FITC (1:200), IA/IE-PE594 (1:200), CD11c-Blue (1:200) (Biolegend, USA) and examined with a BD LSR FortessaX2 flow cytometer. The data were analyzed using the FlowJo10 software.

### ELISA

The assays were carried out according to the instructions of the ELISA kits for HMGB1 (MU30043; Bioswamp, China), heat shock protein (HSP)-60 (MU30603; Bioswamp, China), adenosine triphosphate (ATP) (MU32950; Bioswamp, China), and uric acid (UA) (C012; Nanjing Jiancheng, China).

### Cellular immunofluorescence

For immunofluorescence staining, secondary antibodies with 594 and 488 fluorescence markers were bought from Abbkine, USA. Images were obtained using a 40X confocal laser microscope (Zeiss, USA).

### Protein extraction and western blot analysis

RIPA lysate containing PMSF (Phenylmethanesulfonyl fluoride, 1 mmol·L^−1^) was added to cells for total protein extraction. The proteins were treated with primary antibodies against TLR4, MYD88, COL1A1, RUNX2, and OSX (1:1 000; Abclonal, China); P65 and p-P65 (1:1 000; CST, USA); and GAPDH (1:5 000; Proteintech, USA). The WesternBright ECL HRP substrate kit (Advansta, USA) was used for the visualization of the results.

### Statistical analysis

Statistical analysis was performed with GraphPad Prism version 8.0.2 (GraphPad Software, USA). Results with *P* < 0.05 were considered significant.

## Supplementary information

Supplementary file

## Data Availability

The data supporting the findings of this study are available from the corresponding author upon reasonable request.
