# Supplementary material for: Role of dendritic cells in MYD88-mediated immune recognition and osteoinduction initiated by the implantation of biomaterials
Source: Int J Oral Sci. 2023 Aug 2;15:31. doi: 10.1038/s41368-023-00234-3 (PMC10397189; doi:10.1038/s41368-023-00234-3)
Supplement: Supplementary file 1 — Supplementary file [file 41368_2023_234_MOESM1_ESM.docx]

Supplementary material


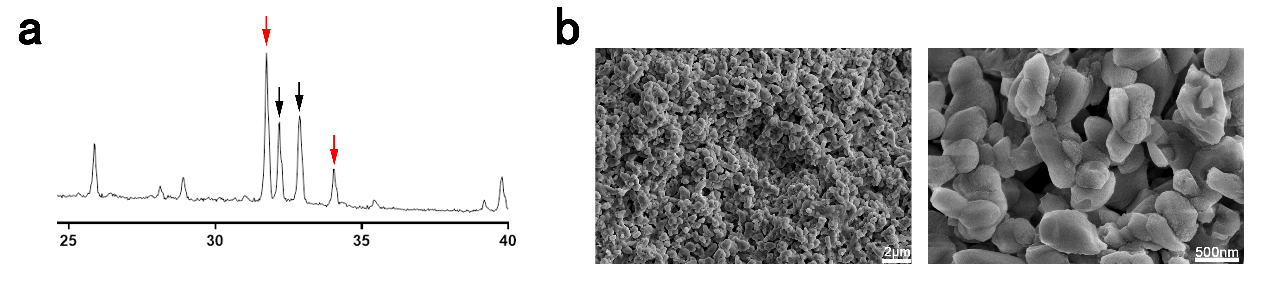


Supplementary Figure 1. Characterization of BCP granules

**a** X-ray diffraction (XRD) of BCP. The characteristic peak of HA and β-TCP were marked by black arrows and red arrows. **b** The scanning electron microscopy (SEM) images of BCP. Magnification were 5k (left) and 30k (right) respectively.


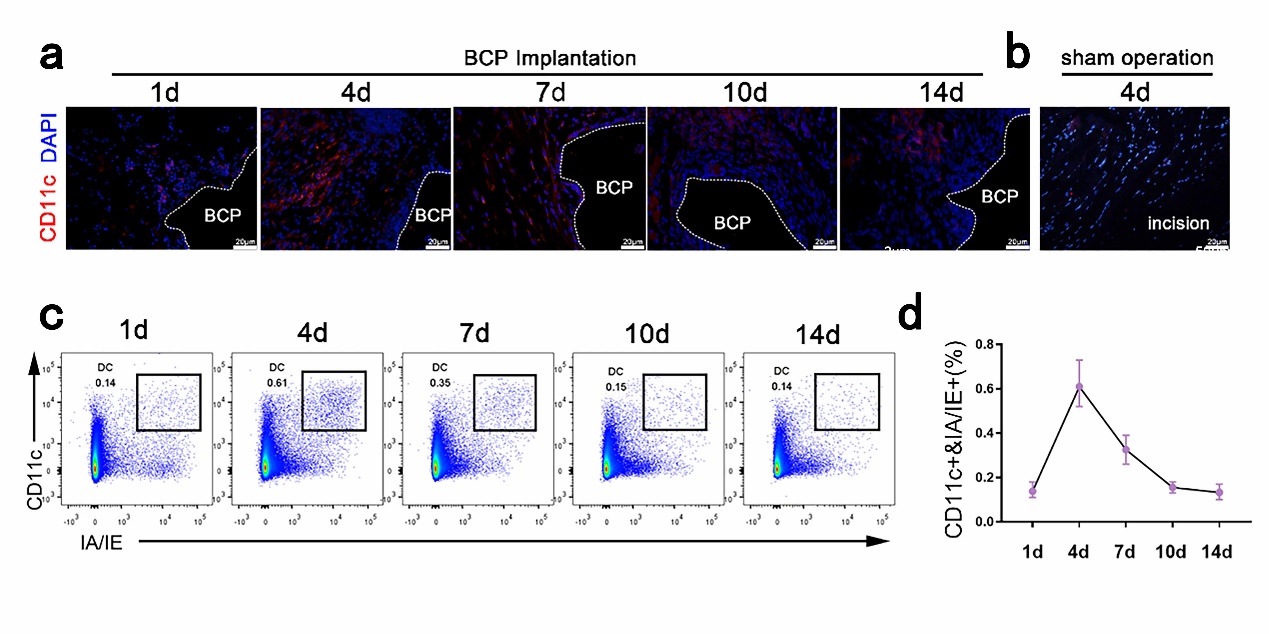


Supplementary Figure 2. Recruitment of DCs around BCP and peak in the number of DCs at 4 days

**a-b** Immunofluorescence staining of CD11c (red) and the nucleus (blue) after BCP implantation **a** or sham operation **b**. **c** Flow cytometry analysis of DCs (CD11c+, IA/IE+) after BCP implantation. **d** Quantification of gating strategy cells in **c** (n = 3).


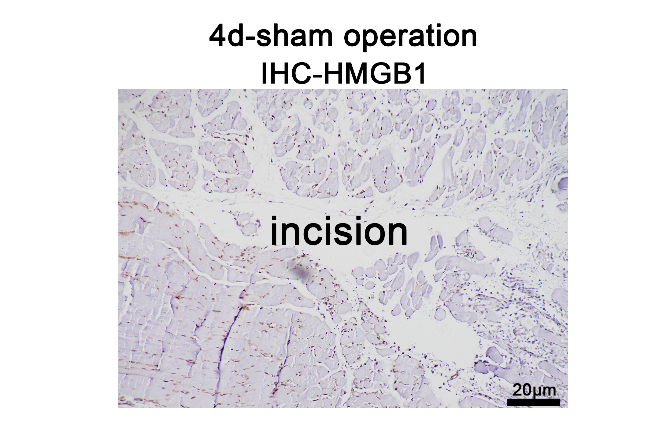


Supplementary Figure 3. On the 4th day after sham surgery, no significant extracellular soluble HMGB1 was detected in the tissues surrounding the incision.


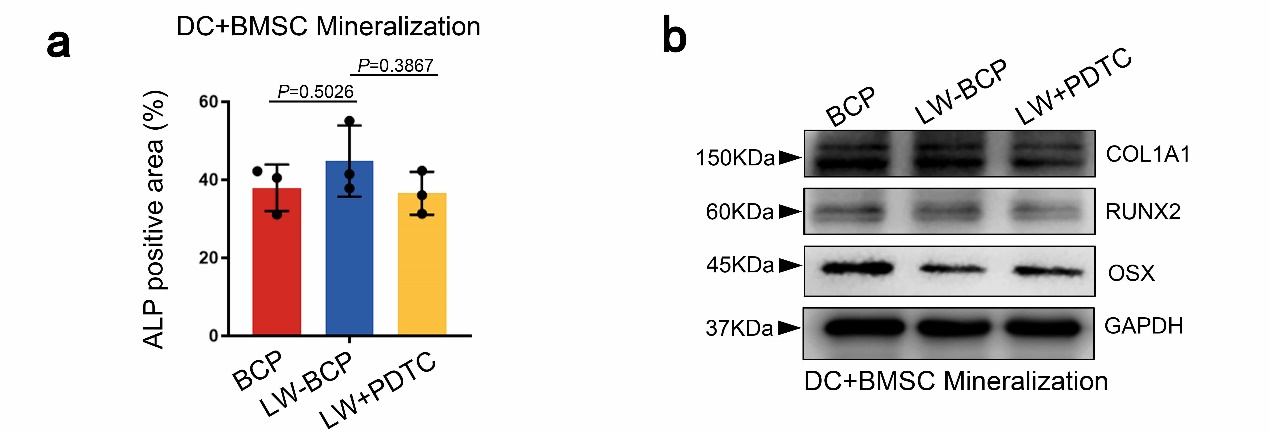


Supplementary Figure 4. No significant effect of DC activation on the mineralization of MSCs

**a** Quantification of ALP staining showing the extent of mineralization in each group. **b** Western blots depicting expression of the mineralized proteins COL1A1, RUNX2, and OSX in each group. BMSCs were treated for 14 days.
